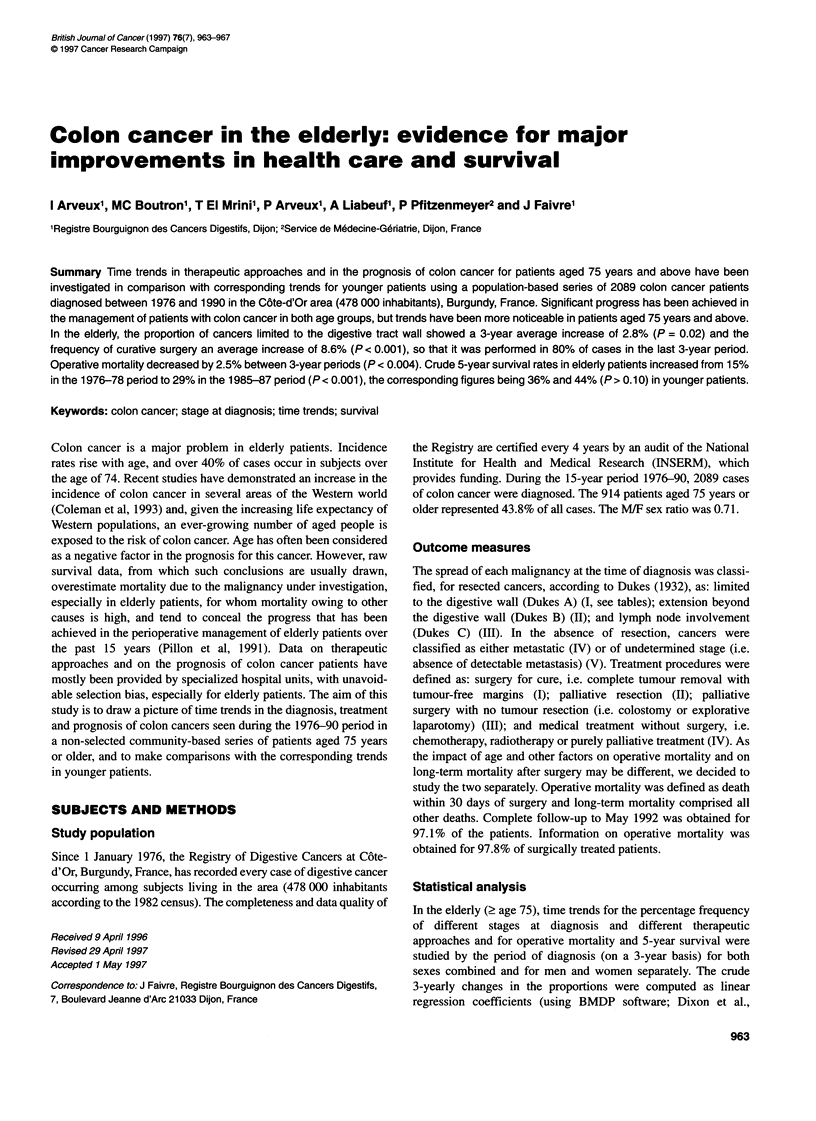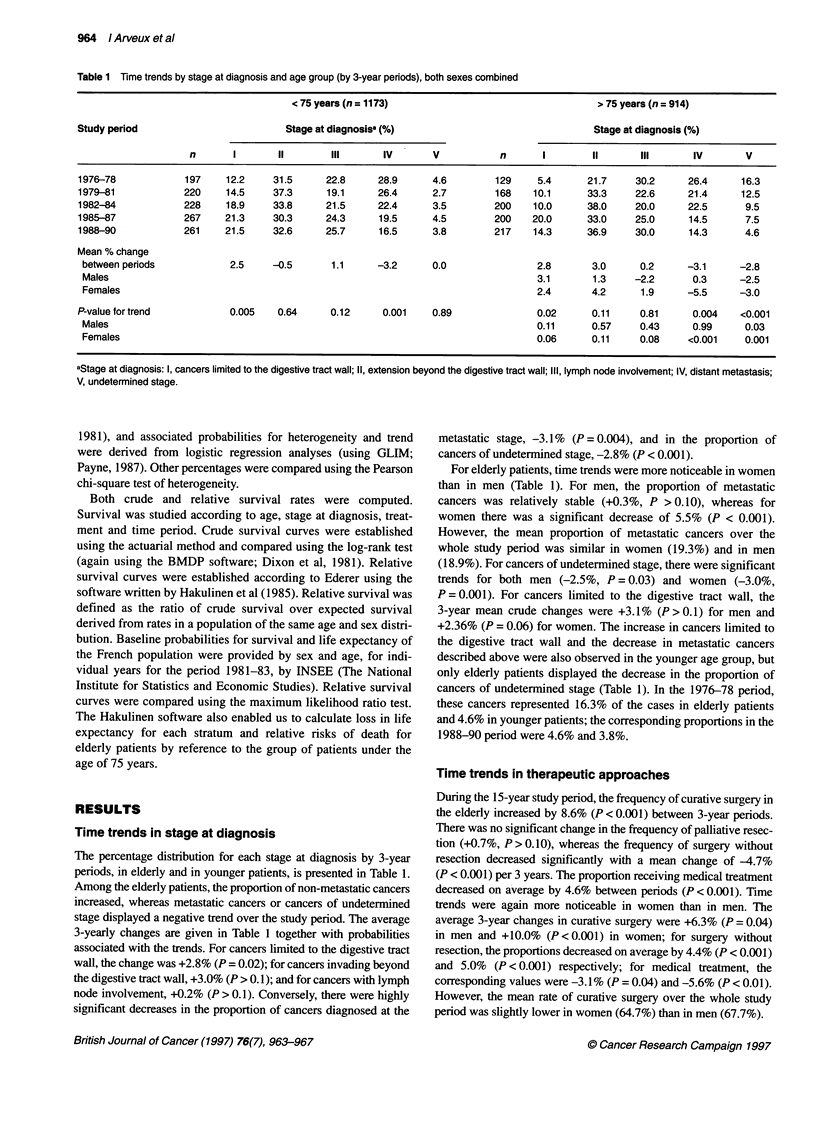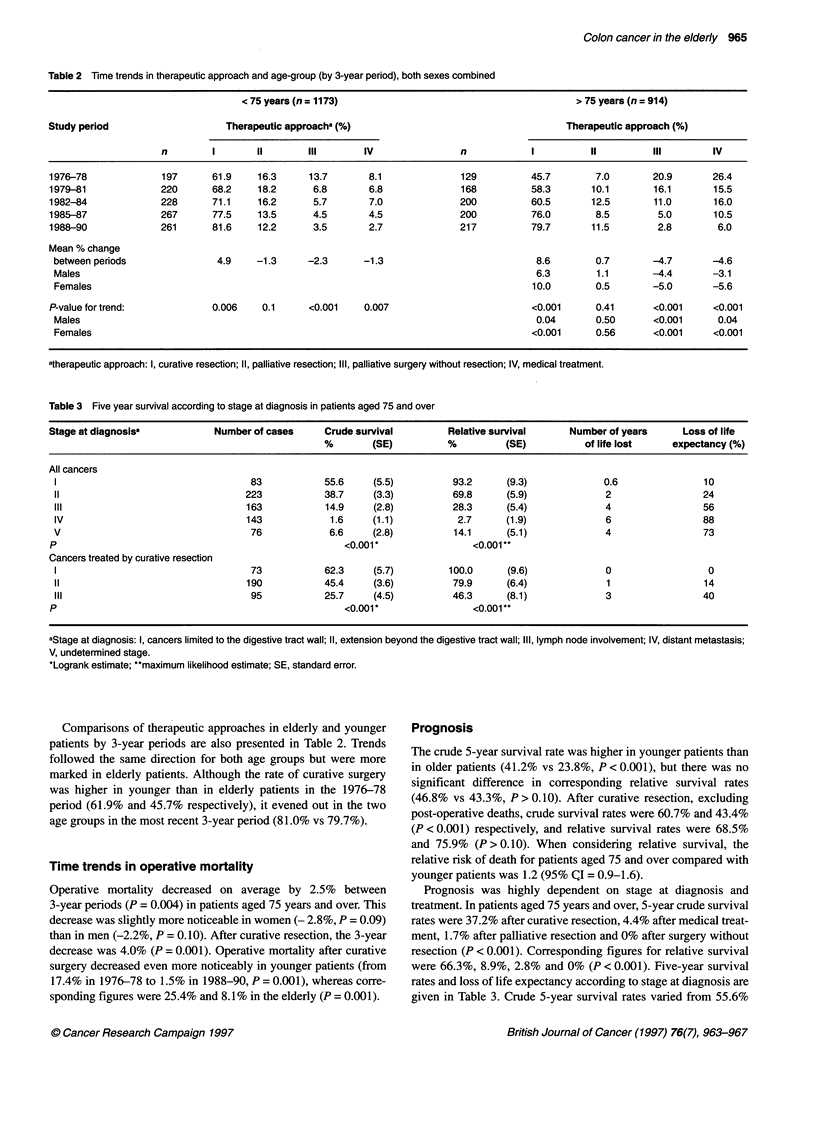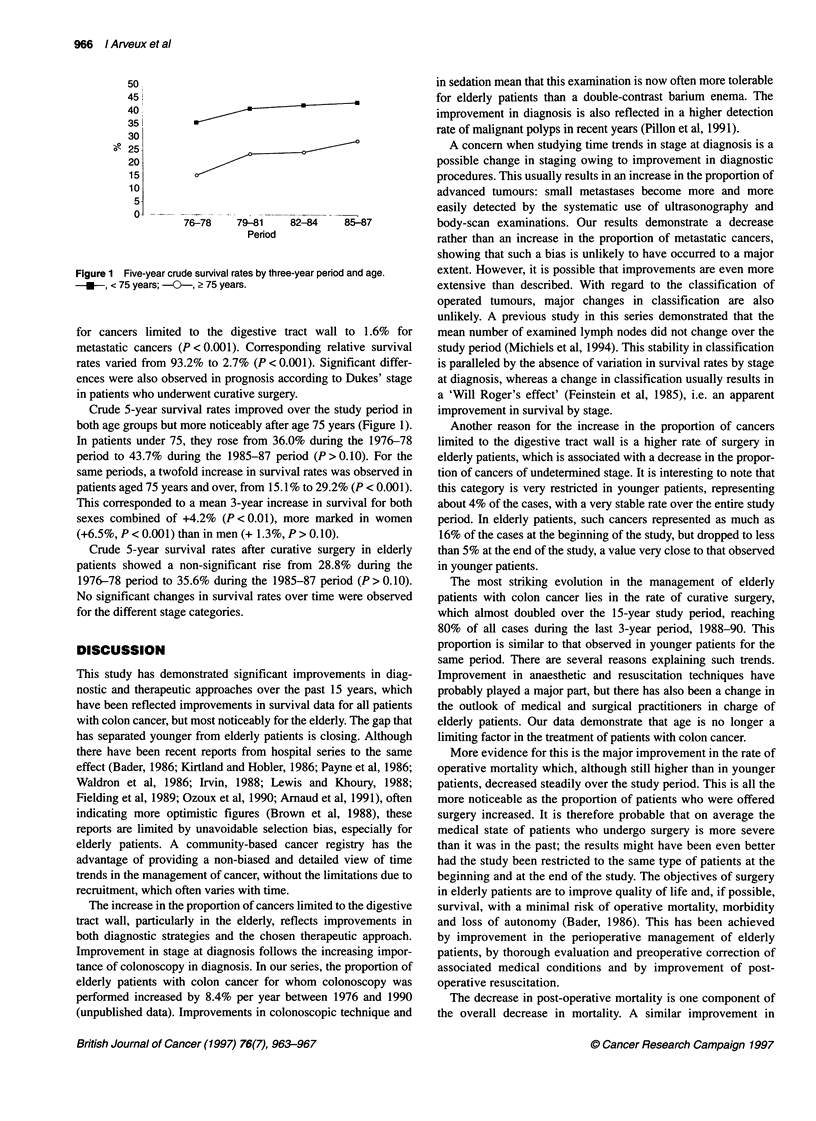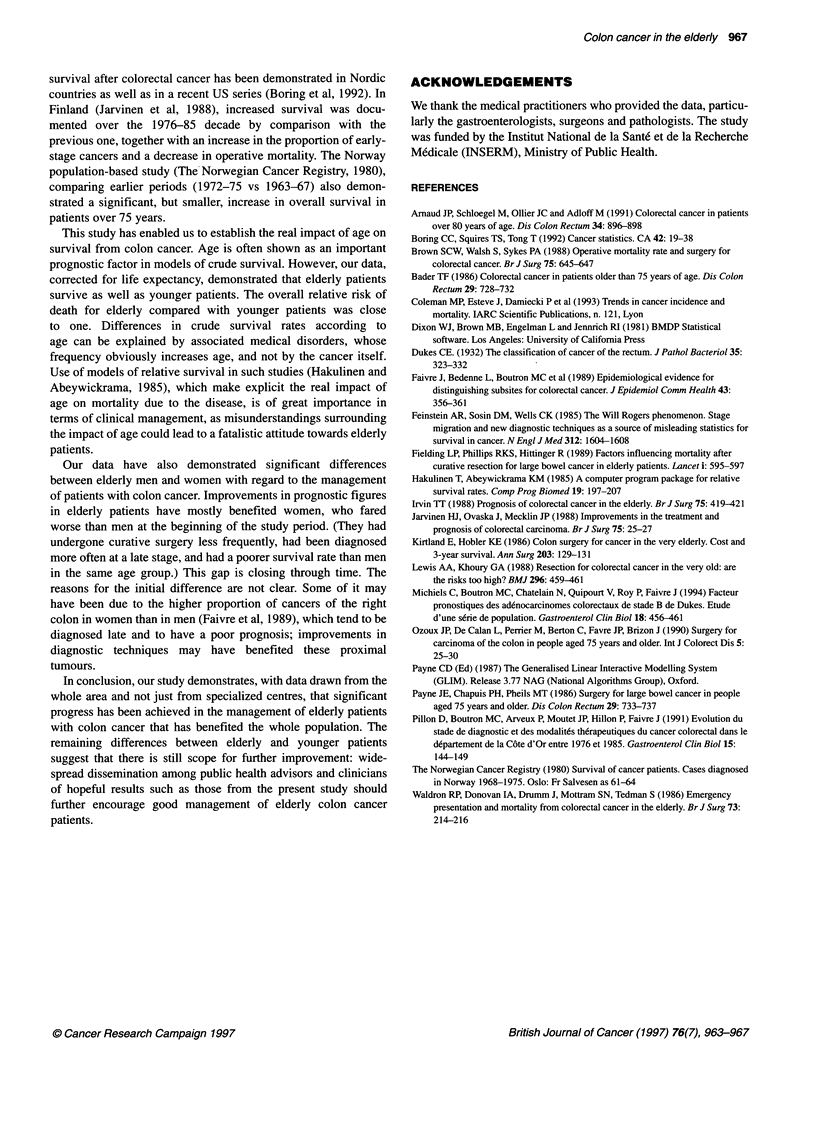# Colon cancer in the elderly: evidence for major improvements in health care and survival.

**DOI:** 10.1038/bjc.1997.492

**Published:** 1997

**Authors:** I. Arveux, M. C. Boutron, T. El Mrini, P. Arveux, A. Liabeuf, P. Pfitzenmeyer, J. Faivre

**Affiliations:** Registre Bourguignon des Cancers Digestifs, Dijon, France.

## Abstract

Time trends in therapeutic approaches and in the prognosis of colon cancer for patients aged 75 years and above have been investigated in comparison with corresponding trends for younger patients using a population-based series of 2089 colon cancer patients diagnosed between 1976 and 1990 in the CÃ´te-d'Or area (478,000 inhabitants), Burgundy, France. Significant progress has been achieved in the management of patients with colon cancer in both age groups, but trends have been more noticeable in patients aged 75 years and above. In the elderly, the proportion of cancers limited to the digestive tract wall showed a 3-year average increase of 2.8% (P = 0.02) and the frequency of curative surgery an average increase of 8.6% (P < 0.001), so that it was performed in 80% of cases in the last 3-year period. Operative mortality decreased by 2.5% between 3-year periods (P < 0.004). Crude 5-year survival rates in elderly patients increased from 15% in the 1976-78 period to 29% in the 1985-87 period (P < 0.001), the corresponding figures being 36% and 44% (P > 0.10) in younger patients.


British Journal of Cancer (1997) 76(7), 963–967
© 1997 Cancer Research Campaign

### Colon cancer in the elderly: evidence for major improvements in health care and survival

I Arveux[1], MC Boutron[1], T El Mrini[1], P Arveux[1], A Liabeuf[1], P Pfitzenmeyer[2] and J Faivre[1]

[1]Registre Bourguignon des Cancers Digestifs, Dijon; [2]Service de Médecine-Gériatrie, Dijon, France

**Summary** Time trends in therapeutic approaches and in the prognosis of colon cancer for patients aged 75 years and above have been investigated in comparison with corresponding trends for younger patients using a population-based series of 2089 colon cancer patients diagnosed between 1976 and 1990 in the Côte-d'Or area (478 000 inhabitants), Burgundy, France. Significant progress has been achieved in the management of patients with colon cancer in both age groups, but trends have been more noticeable in patients aged 75 years and above. In the elderly, the proportion of cancers limited to the digestive tract wall showed a 3-year average increase of 2.8% ($P$ = 0.02) and the frequency of curative surgery an average increase of 8.6% ($P$ < 0.001), so that it was performed in 80% of cases in the last 3-year period. Operative mortality decreased by 2.5% between 3-year periods ($P$ < 0.004). Crude 5-year survival rates in elderly patients increased from 15% in the 1976–78 period to 29% in the 1985–87 period ($P$ < 0.001), the corresponding figures being 36% and 44% ($P$ > 0.10) in younger patients.

**Keywords:** colon cancer; stage at diagnosis; time trends; survival

Colon cancer is a major problem in elderly patients. Incidence rates rise with age, and over 40% of cases occur in subjects over the age of 74. Recent studies have demonstrated an increase in the incidence of colon cancer in several areas of the Western world (Coleman et al, 1993) and, given the increasing life expectancy of Western populations, an ever-growing number of aged people is exposed to the risk of colon cancer. Age has often been considered as a negative factor in the prognosis for this cancer. However, raw survival data, from which such conclusions are usually drawn, overestimate mortality due to the malignancy under investigation, especially in elderly patients, for whom mortality owing to other causes is high, and tend to conceal the progress that has been achieved in the perioperative management of elderly patients over the past 15 years (Pillon et al, 1991). Data on therapeutic approaches and on the prognosis of colon cancer patients have mostly been provided by specialized hospital units, with unavoidable selection bias, especially for elderly patients. The aim of this study is to draw a picture of time trends in the diagnosis, treatment and prognosis of colon cancers seen during the 1976–90 period in a non-selected community-based series of patients aged 75 years or older, and to make comparisons with the corresponding trends in younger patients.

### SUBJECTS AND METHODS

### Study population

Since 1 January 1976, the Registry of Digestive Cancers at Côte-d'Or, Burgundy, France, has recorded every case of digestive cancer occurring among subjects living in the area (478 000 inhabitants according to the 1982 census). The completeness and data quality of the Registry are certified every 4 years by an audit of the National Institute for Health and Medical Research (INSERM), which provides funding. During the 15-year period 1976–90, 2089 cases of colon cancer were diagnosed. The 914 patients aged 75 years or older represented 43.8% of all cases. The M/F sex ratio was 0.71.

*Received 9 April 1996*
*Revised 29 April 1997*
*Accepted 1 May 1997*

*Correspondence to:* J Faivre, Registre Bourguignon des Cancers Digestifs, 7, Boulevard Jeanne d'Arc 21033 Dijon, France

### Outcome measures

The spread of each malignancy at the time of diagnosis was classified, for resected cancers, according to Dukes (1932), as: limited to the digestive wall (Dukes A) (I, see tables); extension beyond the digestive wall (Dukes B) (II); and lymph node involvement (Dukes C) (III). In the absence of resection, cancers were classified as either metastatic (IV) or of undetermined stage (i.e. absence of detectable metastasis) (V). Treatment procedures were defined as: surgery for cure, i.e. complete tumour removal with tumour-free margins (I); palliative resection (II); palliative surgery with no tumour resection (i.e. colostomy or explorative laparotomy) (III); and medical treatment without surgery, i.e. chemotherapy, radiotherapy or purely palliative treatment (IV). As the impact of age and other factors on operative mortality and on long-term mortality after surgery may be different, we decided to study the two separately. Operative mortality was defined as death within 30 days of surgery and long-term mortality comprised all other deaths. Complete follow-up to May 1992 was obtained for 97.1% of the patients. Information on operative mortality was obtained for 97.8% of surgically treated patients.

### Statistical analysis

In the elderly (≥ age 75), time trends for the percentage frequency of different stages at diagnosis and different therapeutic approaches and for operative mortality and 5-year survival were studied by the period of diagnosis (on a 3-year basis) for both sexes combined and for men and women separately. The crude 3-yearly changes in the proportions were computed as linear regression coefficients (using BMDP software; Dixon et al.,

Table 1 Time trends by stage at diagnosis and age group (by 3-year periods), both sexes combined

| Study period | < 75 years (n = 1173) | | | | | | > 75 years (n = 914) | | | | | |
|---|---|---|---|---|---|---|---|---|---|---|---|---|
| | | Stage at diagnosis[a] (%) | | | | | | Stage at diagnosis (%) | | | | |
| | n | I | II | III | IV | V | n | I | II | III | IV | V |
| 1976–78 | 197 | 12.2 | 31.5 | 22.8 | 28.9 | 4.6 | 129 | 5.4 | 21.7 | 30.2 | 26.4 | 16.3 |
| 1979–81 | 220 | 14.5 | 37.3 | 19.1 | 26.4 | 2.7 | 168 | 10.1 | 33.3 | 22.6 | 21.4 | 12.5 |
| 1982–84 | 228 | 18.9 | 33.8 | 21.5 | 22.4 | 3.5 | 200 | 10.0 | 38.0 | 20.0 | 22.5 | 9.5 |
| 1985–87 | 267 | 21.3 | 30.3 | 24.3 | 19.5 | 4.5 | 200 | 20.0 | 33.0 | 25.0 | 14.5 | 7.5 |
| 1988–90 | 261 | 21.5 | 32.6 | 25.7 | 16.5 | 3.8 | 217 | 14.3 | 36.9 | 30.0 | 14.3 | 4.6 |
| Mean % change between periods | | 2.5 | –0.5 | 1.1 | –3.2 | 0.0 | | 2.8 | 3.0 | 0.2 | –3.1 | –2.8 |
| Males | | | | | | | | 3.1 | 1.3 | –2.2 | 0.3 | –2.5 |
| Females | | | | | | | | 2.4 | 4.2 | 1.9 | –5.5 | –3.0 |
| P-value for trend | | 0.005 | 0.64 | 0.12 | 0.001 | 0.89 | | 0.02 | 0.11 | 0.81 | 0.004 | <0.001 |
| Males | | | | | | | | 0.11 | 0.57 | 0.43 | 0.99 | 0.03 |
| Females | | | | | | | | 0.06 | 0.11 | 0.08 | <0.001 | 0.001 |

[a]Stage at diagnosis: I, cancers limited to the digestive tract wall; II, extension beyond the digestive tract wall; III, lymph node involvement; IV, distant metastasis; V, undetermined stage.

1981), and associated probabilities for heterogeneity and trend were derived from logistic regression analyses (using GLIM; Payne, 1987). Other percentages were compared using the Pearson chi-square test of heterogeneity.

Both crude and relative survival rates were computed. Survival was studied according to age, stage at diagnosis, treatment and time period. Crude survival curves were established using the actuarial method and compared using the log-rank test (again using the BMDP software; Dixon et al, 1981). Relative survival curves were established according to Ederer using the software written by Hakulinen et al (1985). Relative survival was defined as the ratio of crude survival over expected survival derived from rates in a population of the same age and sex distribution. Baseline probabilities for survival and life expectancy of the French population were provided by sex and age, for individual years for the period 1981–83, by INSEE (The National Institute for Statistics and Economic Studies). Relative survival curves were compared using the maximum likelihood ratio test. The Hakulinen software also enabled us to calculate loss in life expectancy for each stratum and relative risks of death for elderly patients by reference to the group of patients under the age of 75 years.

### RESULTS

### Time trends in stage at diagnosis

The percentage distribution for each stage at diagnosis by 3-year periods, in elderly and in younger patients, is presented in Table 1. Among the elderly patients, the proportion of non-metastatic cancers increased, whereas metastatic cancers or cancers of undetermined stage displayed a negative trend over the study period. The average 3-yearly changes are given in Table 1 together with probabilities associated with the trends. For cancers limited to the digestive tract wall, the change was +2.8% ($P = 0.02$); for cancers invading beyond the digestive tract wall, +3.0% ($P > 0.1$); and for cancers with lymph node involvement, +0.2% ($P > 0.1$). Conversely, there were highly significant decreases in the proportion of cancers diagnosed at the metastatic stage, –3.1% ($P = 0.004$), and in the proportion of cancers of undetermined stage, –2.8% ($P < 0.001$).

For elderly patients, time trends were more noticeable in women than in men (Table 1). For men, the proportion of metastatic cancers was relatively stable (+0.3%, $P > 0.10$), whereas for women there was a significant decrease of 5.5% ($P < 0.001$). However, the mean proportion of metastatic cancers over the whole study period was similar in women (19.3%) and in men (18.9%). For cancers of undetermined stage, there were significant trends for both men (–2.5%, $P = 0.03$) and women (–3.0%, $P = 0.001$). For cancers limited to the digestive tract wall, the 3-year mean crude changes were +3.1% ($P > 0.1$) for men and +2.36% ($P = 0.06$) for women. The increase in cancers limited to the digestive tract wall and the decrease in metastatic cancers described above were also observed in the younger age group, but only elderly patients displayed the decrease in the proportion of cancers of undetermined stage (Table 1). In the 1976–78 period, these cancers represented 16.3% of the cases in elderly patients and 4.6% in younger patients; the corresponding proportions in the 1988–90 period were 4.6% and 3.8%.

### Time trends in therapeutic approaches

During the 15-year study period, the frequency of curative surgery in the elderly increased by 8.6% ($P < 0.001$) between 3-year periods. There was no significant change in the frequency of palliative resection (+0.7%, $P > 0.10$), whereas the frequency of surgery without resection decreased significantly with a mean change of –4.7% ($P < 0.001$) per 3 years. The proportion receiving medical treatment decreased on average by 4.6% between periods ($P < 0.001$). Time trends were again more noticeable in women than in men. The average 3-year changes in curative surgery were +6.3% ($P = 0.04$) in men and +10.0% ($P < 0.001$) in women; for surgery without resection, the proportions decreased on average by 4.4% ($P < 0.001$) and 5.0% ($P < 0.001$) respectively; for medical treatment, the corresponding values were –3.1% ($P = 0.04$) and –5.6% ($P < 0.01$). However, the mean rate of curative surgery over the whole study period was slightly lower in women (64.7%) than in men (67.7%).

© Cancer Research Campaign 1997

**Table 2** Time trends in therapeutic approach and age-group (by 3-year period), both sexes combined

| Study period | < 75 years ($n$ = 1173) | | | | | > 75 years ($n$ = 914) | | | | |
|---|---|---|---|---|---|---|---|---|---|---|
| | | Therapeutic approach[a] (%) | | | | | Therapeutic approach (%) | | | |
| | $n$ | I | II | III | IV | $n$ | I | II | III | IV |
| 1976–78 | 197 | 61.9 | 16.3 | 13.7 | 8.1 | 129 | 45.7 | 7.0 | 20.9 | 26.4 |
| 1979–81 | 220 | 68.2 | 18.2 | 6.8 | 6.8 | 168 | 58.3 | 10.1 | 16.1 | 15.5 |
| 1982–84 | 228 | 71.1 | 16.2 | 5.7 | 7.0 | 200 | 60.5 | 12.5 | 11.0 | 16.0 |
| 1985–87 | 267 | 77.5 | 13.5 | 4.5 | 4.5 | 200 | 76.0 | 8.5 | 5.0 | 10.5 |
| 1988–90 | 261 | 81.6 | 12.2 | 3.5 | 2.7 | 217 | 79.7 | 11.5 | 2.8 | 6.0 |
| Mean % change | | | | | | | | | | |
| between periods | | 4.9 | –1.3 | –2.3 | –1.3 | | 8.6 | 0.7 | –4.7 | –4.6 |
| Males | | | | | | | 6.3 | 1.1 | –4.4 | –3.1 |
| Females | | | | | | | 10.0 | 0.5 | –5.0 | –5.6 |
| $P$-value for trend: | | 0.006 | 0.1 | <0.001 | 0.007 | | <0.001 | 0.41 | <0.001 | <0.001 |
| Males | | | | | | | 0.04 | 0.50 | <0.001 | 0.04 |
| Females | | | | | | | <0.001 | 0.56 | <0.001 | <0.001 |

[a]therapeutic approach: I, curative resection; II, palliative resection; III, palliative surgery without resection; IV, medical treatment.

**Table 3** Five year survival according to stage at diagnosis in patients aged 75 and over

| Stage at diagnosis[a] | Number of cases | Crude survival % | (SE) | Relative survival % | (SE) | Number of years of life lost | Loss of life expectancy (%) |
|---|---|---|---|---|---|---|---|
| All cancers | | | | | | | |
| I | 83 | 55.6 | (5.5) | 93.2 | (9.3) | 0.6 | 10 |
| II | 223 | 38.7 | (3.3) | 69.8 | (5.9) | 2 | 24 |
| III | 163 | 14.9 | (2.8) | 28.3 | (5.4) | 4 | 56 |
| IV | 143 | 1.6 | (1.1) | 2.7 | (1.9) | 6 | 88 |
| V | 76 | 6.6 | (2.8) | 14.1 | (5.1) | 4 | 73 |
| $P$ | | <0.001* | | <0.001** | | | |
| Cancers treated by curative resection | | | | | | | |
| I | 73 | 62.3 | (5.7) | 100.0 | (9.6) | 0 | 0 |
| II | 190 | 45.4 | (3.6) | 79.9 | (6.4) | 1 | 14 |
| III | 95 | 25.7 | (4.5) | 46.3 | (8.1) | 3 | 40 |
| $P$ | | <0.001* | | <0.001** | | | |

[a]Stage at diagnosis: I, cancers limited to the digestive tract wall; II, extension beyond the digestive tract wall; III, lymph node involvement; IV, distant metastasis; V, undetermined stage.
*Logrank estimate; **maximum likelihood estimate; SE, standard error.

Comparisons of therapeutic approaches in elderly and younger patients by 3-year periods are also presented in Table 2. Trends followed the same direction for both age groups but were more marked in elderly patients. Although the rate of curative surgery was higher in younger than in elderly patients in the 1976–78 period (61.9% and 45.7% respectively), it evened out in the two age groups in the most recent 3-year period (81.0% vs 79.7%).

### Time trends in operative mortality

Operative mortality decreased on average by 2.5% between 3-year periods ($P$ = 0.004) in patients aged 75 years and over. This decrease was slightly more noticeable in women (– 2.8%, $P$ = 0.09) than in men (–2.2%, $P$ = 0.10). After curative resection, the 3-year decrease was 4.0% ($P$ = 0.001). Operative mortality after curative surgery decreased even more noticeably in younger patients (from 17.4% in 1976–78 to 1.5% in 1988–90, $P$ = 0.001), whereas corresponding figures were 25.4% and 8.1% in the elderly ($P$ = 0.001).

### Prognosis

The crude 5-year survival rate was higher in younger patients than in older patients (41.2% vs 23.8%, $P$ < 0.001), but there was no significant difference in corresponding relative survival rates (46.8% vs 43.3%, $P$ > 0.10). After curative resection, excluding post-operative deaths, crude survival rates were 60.7% and 43.4% ($P$ < 0.001) respectively, and relative survival rates were 68.5% and 75.9% ($P$ > 0.10). When considering relative survival, the relative risk of death for patients aged 75 and over compared with younger patients was 1.2 (95% CI = 0.9–1.6).

Prognosis was highly dependent on stage at diagnosis and treatment. In patients aged 75 years and over, 5-year crude survival rates were 37.2% after curative resection, 4.4% after medical treatment, 1.7% after palliative resection and 0% after surgery without resection ($P$ < 0.001). Corresponding figures for relative survival were 66.3%, 8.9%, 2.8% and 0% ($P$ < 0.001). Five-year survival rates and loss of life expectancy according to stage at diagnosis are given in Table 3. Crude 5-year survival rates varied from 55.6%

**Figure 1** Five-year crude survival rates by three-year period and age. —■—, < 75 years; —○—, ≥ 75 years.

for cancers limited to the digestive tract wall to 1.6% for metastatic cancers ($P < 0.001$). Corresponding relative survival rates varied from 93.2% to 2.7% ($P < 0.001$). Significant differences were also observed in prognosis according to Dukes' stage in patients who underwent curative surgery.

Crude 5-year survival rates improved over the study period in both age groups but more noticeably after age 75 years (Figure 1). In patients under 75, they rose from 36.0% during the 1976–78 period to 43.7% during the 1985–87 period ($P > 0.10$). For the same periods, a twofold increase in survival rates was observed in patients aged 75 years and over, from 15.1% to 29.2% ($P < 0.001$). This corresponded to a mean 3-year increase in survival for both sexes combined of +4.2% ($P < 0.01$), more marked in women (+6.5%, $P < 0.001$) than in men (+ 1.3%, $P > 0.10$).

Crude 5-year survival rates after curative surgery in elderly patients showed a non-significant rise from 28.8% during the 1976–78 period to 35.6% during the 1985–87 period ($P > 0.10$). No significant changes in survival rates over time were observed for the different stage categories.

### DISCUSSION

This study has demonstrated significant improvements in diagnostic and therapeutic approaches over the past 15 years, which have been reflected improvements in survival data for all patients with colon cancer, but most noticeably for the elderly. The gap that has separated younger from elderly patients is closing. Although there have been recent reports from hospital series to the same effect (Bader, 1986; Kirtland and Hobler, 1986; Payne et al, 1986; Waldron et al, 1986; Irvin, 1988; Lewis and Khoury, 1988; Fielding et al, 1989; Ozoux et al, 1990; Arnaud et al, 1991), often indicating more optimistic figures (Brown et al, 1988), these reports are limited by unavoidable selection bias, especially for elderly patients. A community-based cancer registry has the advantage of providing a non-biased and detailed view of time trends in the management of cancer, without the limitations due to recruitment, which often varies with time.

The increase in the proportion of cancers limited to the digestive tract wall, particularly in the elderly, reflects improvements in both diagnostic strategies and the chosen therapeutic approach. Improvement in stage at diagnosis follows the increasing importance of colonoscopy in diagnosis. In our series, the proportion of elderly patients with colon cancer for whom colonoscopy was performed increased by 8.4% per year between 1976 and 1990 (unpublished data). Improvements in colonoscopic technique and in sedation mean that this examination is now often more tolerable for elderly patients than a double-contrast barium enema. The improvement in diagnosis is also reflected in a higher detection rate of malignant polyps in recent years (Pillon et al, 1991).

A concern when studying time trends in stage at diagnosis is a possible change in staging owing to improvement in diagnostic procedures. This usually results in an increase in the proportion of advanced tumours: small metastases become more and more easily detected by the systematic use of ultrasonography and body-scan examinations. Our results demonstrate a decrease rather than an increase in the proportion of metastatic cancers, showing that such a bias is unlikely to have occurred to a major extent. However, it is possible that improvements are even more extensive than described. With regard to the classification of operated tumours, major changes in classification are also unlikely. A previous study in this series demonstrated that the mean number of examined lymph nodes did not change over the study period (Michiels et al, 1994). This stability in classification is paralleled by the absence of variation in survival rates by stage at diagnosis, whereas a change in classification usually results in a 'Will Roger's effect' (Feinstein et al, 1985), i.e. an apparent improvement in survival by stage.

Another reason for the increase in the proportion of cancers limited to the digestive tract wall is a higher rate of surgery in elderly patients, which is associated with a decrease in the proportion of cancers of undetermined stage. It is interesting to note that this category is very restricted in younger patients, representing about 4% of the cases, with a very stable rate over the entire study period. In elderly patients, such cancers represented as much as 16% of the cases at the beginning of the study, but dropped to less than 5% at the end of the study, a value very close to that observed in younger patients.

The most striking evolution in the management of elderly patients with colon cancer lies in the rate of curative surgery, which almost doubled over the 15-year study period, reaching 80% of all cases during the last 3-year period, 1988–90. This proportion is similar to that observed in younger patients for the same period. There are several reasons explaining such trends. Improvement in anaesthetic and resuscitation techniques have probably played a major part, but there has also been a change in the outlook of medical and surgical practitioners in charge of elderly patients. Our data demonstrate that age is no longer a limiting factor in the treatment of patients with colon cancer.

More evidence for this is the major improvement in the rate of operative mortality which, although still higher than in younger patients, decreased steadily over the study period. This is all the more noticeable as the proportion of patients who were offered surgery increased. It is therefore probable that on average the medical state of patients who undergo surgery is more severe than it was in the past; the results might have been even better had the study been restricted to the same type of patients at the beginning and at the end of the study. The objectives of surgery in elderly patients are to improve quality of life and, if possible, survival, with a minimal risk of operative mortality, morbidity and loss of autonomy (Bader, 1986). This has been achieved by improvement in the perioperative management of elderly patients, by thorough evaluation and preoperative correction of associated medical conditions and by improvement of post-operative resuscitation.

The decrease in post-operative mortality is one component of the overall decrease in mortality. A similar improvement in

© Cancer Research Campaign 1997

survival after colorectal cancer has been demonstrated in Nordic countries as well as in a recent US series (Boring et al, 1992). In Finland (Jarvinen et al, 1988), increased survival was documented over the 1976–85 decade by comparison with the previous one, together with an increase in the proportion of early-stage cancers and a decrease in operative mortality. The Norway population-based study (The Norwegian Cancer Registry, 1980), comparing earlier periods (1972–75 vs 1963–67) also demonstrated a significant, but smaller, increase in overall survival in patients over 75 years.

This study has enabled us to establish the real impact of age on survival from colon cancer. Age is often shown as an important prognostic factor in models of crude survival. However, our data, corrected for life expectancy, demonstrated that elderly patients survive as well as younger patients. The overall relative risk of death for elderly compared with younger patients was close to one. Differences in crude survival rates according to age can be explained by associated medical disorders, whose frequency obviously increases age, and not by the cancer itself. Use of models of relative survival in such studies (Hakulinen and Abeywickrama, 1985), which make explicit the real impact of age on mortality due to the disease, is of great importance in terms of clinical management, as misunderstandings surrounding the impact of age could lead to a fatalistic attitude towards elderly patients.

Our data have also demonstrated significant differences between elderly men and women with regard to the management of patients with colon cancer. Improvements in prognostic figures in elderly patients have mostly benefited women, who fared worse than men at the beginning of the study period. (They had undergone curative surgery less frequently, had been diagnosed more often at a late stage, and had a poorer survival rate than men in the same age group.) This gap is closing through time. The reasons for the initial difference are not clear. Some of it may have been due to the higher proportion of cancers of the right colon in women than in men (Faivre et al, 1989), which tend to be diagnosed late and to have a poor prognosis; improvements in diagnostic techniques may have benefited these proximal tumours.

In conclusion, our study demonstrates, with data drawn from the whole area and not just from specialized centres, that significant progress has been achieved in the management of elderly patients with colon cancer that has benefited the whole population. The remaining differences between elderly and younger patients suggest that there is still scope for further improvement: widespread dissemination among public health advisors and clinicians of hopeful results such as those from the present study should further encourage good management of elderly colon cancer patients.

### ACKNOWLEDGEMENTS

We thank the medical practitioners who provided the data, particularly the gastroenterologists, surgeons and pathologists. The study was funded by the Institut National de la Santé et de la Recherche Médicale (INSERM), Ministry of Public Health.

### REFERENCES

Arnaud JP, Schloegel M, Ollier JC and Adloff M (1991) Colorectal cancer in patients over 80 years of age. *Dis Colon Rectum* **34**: 896–898

Boring CC, Squires TS, Tong T (1992) Cancer statistics. CA **42**: 19–38

Brown SCW, Walsh S, Sykes PA (1988) Operative mortality rate and surgery for colorectal cancer. *Br J Surg* **75**: 645–647

Bader TF (1986) Colorectal cancer in patients older than 75 years of age. *Dis Colon Rectum* **29**: 728–732

Coleman MP, Esteve J, Damiecki P et al (1993) Trends in cancer incidence and mortality. IARC Scientific Publications, n. 121, Lyon

Dixon WJ, Brown MB, Engelman L and Jennrich RI (1981) BMDP Statistical software. Los Angeles: University of California Press

Dukes CE. (1932) The classification of cancer of the rectum. *J Pathol Bacteriol* **35**: 323–332

Faivre J, Bedenne L, Boutron MC et al (1989) Epidemiological evidence for distinguishing subsites for colorectal cancer. *J Epidemiol Comm Health* **43**: 356–361

Feinstein AR, Sosin DM, Wells CK (1985) The Will Rogers phenomenon. Stage migration and new diagnostic techniques as a source of misleading statistics for survival in cancer. *N Engl J Med* **312**: 1604–1608

Fielding LP, Phillips RKS, Hittinger R (1989) Factors influencing mortality after curative resection for large bowel cancer in elderly patients. *Lancet* **i**: 595–597

Hakulinen T, Abeywickrama KM (1985) A computer program package for relative survival rates. *Comp Prog Biomed* **19**: 197–207

Irvin TT (1988) Prognosis of colorectal cancer in the elderly. *Br J Surg* **75**: 419–421

Jarvinen HJ, Ovaska J, Mecklin JP (1988) Improvements in the treatment and prognosis of colorectal carcinoma. *Br J Surg* **75**: 25–27

Kirtland E, Hobler KE (1986) Colon surgery for cancer in the very elderly. Cost and 3-year survival. *Ann Surg* **203**: 129–131

Lewis AA, Khoury GA (1988) Resection for colorectal cancer in the very old: are the risks too high? *BMJ* **296**: 459–461

Michiels C, Boutron MC, Chatelain N, Quipourt V, Roy P, Faivre J (1994) Facteur pronostiques des adénocarcinomes colorectaux de stade B de Dukes. Etude d'une série de population. *Gastroenterol Clin Biol* **18**: 456–461

Ozoux JP, De Calan L, Perrier M, Berton C, Favre JP, Brizon J (1990) Surgery for carcinoma of the colon in people aged 75 years and older. Int J Colorect Dis **5**: 25–30

Payne CD (Ed) (1987) The Generalised Linear Interactive Modelling System (GLIM). Release 3.77 NAG (National Algorithms Group), Oxford.

Payne JE, Chapuis PH, Pheils MT (1986) Surgery for large bowel cancer in people aged 75 years and older. *Dis Colon Rectum* **29**: 733–737

Pillon D, Boutron MC, Arveux P, Moutet JP, Hillon P, Faivre J (1991) Evolution du stade de diagnostic et des modalités thérapeutiques du cancer colorectal dans le département de la Côte d'Or entre 1976 et 1985. *Gastroenterol Clin Biol* **15**: 144–149

The Norwegian Cancer Registry (1980) Survival of cancer patients. Cases diagnosed in Norway 1968–1975. Oslo: Fr Salvesen as 61–64

Waldron RP, Donovan IA, Drumm J, Mottram SN, Tedman S (1986) Emergency presentation and mortality from colorectal cancer in the elderly. *Br J Surg* **73**: 214–216

© Cancer Research Campaign 1997